# Effects of the immunoglobulin/histamine complex on panic disorder concurrent with chronic spontaneous urticaria: a case report

**DOI:** 10.1186/s13256-023-03937-7

**Published:** 2023-07-28

**Authors:** Hyuk Soon Kim, Geunwoong Noh

**Affiliations:** 1 Department of Biomedical Sciences, College of Natural Science and Department of Health Sciences, The Graduate School of Dong-A University, Busan, South Korea; 2 Department of Allergy and Clinical Immunology, Cheju Halla General Hospital, Doreongno 65, Jeju-si, Jeju Republic of Korea

**Keywords:** Chronic urticaria, Panic disorder, Depression, Anxiety, Immunoglobulin/histamine complex, Case report

## Abstract

**Background:**

Panic disorder and panic attacks are two of the most common problems in psychiatry. A psychoimmunological correlation between allergic diseases and panic disorder has been strongly suggested. Histamine H1 receptor antagonists have been suggested as alternative drugs for the treatment of panic disorder. Chronic spontaneous urticaria (CSU) and panic disorder improved simultaneously with selective serotonin reuptake inhibitor antidepressants. Panic disorder has also been treated with the antihistamine chlorpheniramine. The immunoglobulin/histamine complex is a histamine-fixed immunoglobulin preparation that was reported to be effective in treating CSU. This case report describes the successful treatment of a patient with concomitant panic disorder and CSU for 23 years using immunoglobulin/histamine complex therapy.

**Case presentation:**

This report describes a 52-year-old female Korean patient who suffered from CSU with panic disorder for 23 years. Basic allergy tests (blood tests and skin prick tests) were conducted before and after treatment for the evaluation of allergic conditions. A multiple allergosorbent test (MAST) for the detection of allergen-specific IgE levels was also performed. The clinical severity of CSU was evaluated using the urticaria severity score system. Diagnostic interviews systematically assessed the diagnostic criteria outlined by the DSM-V, and the patient was evaluated before, during and after treatment using the Beck Depression Inventory (BDI-2) for depression, the State-Trait Anxiety Inventory (STAI) for anxiety and the Beck Hopelessness Score (BHS) for hopelessness. The patient received 2 ml of Histobulin™ (12 mg human immunoglobulin/0.15 µg histamine complex) once a week by subcutaneous injection for the treatment of CSU. Initial improvement of CSU was achieved after the third injection. After the twenty-seventh injection of Histobulin™, she showed no symptoms or signs and ceased allergic medication use. With the remission of CSU, allergic rhinitis was also completely resolved. The frequency of the common cold was significantly decreased during and after treatment. The medication frequency and development of clinical manifestations of panic disorder changed in parallel with the clinical severity of CSU. Moreover, the patient exhibited no clinical manifestations and ceased medication for panic disorder and sleeping pills for insomnia simultaneously with the remission of CSU. In the psychological evaluation, the BDI, STAI and BHS scores improved accordingly.

**Conclusions:**

The immunoglobulin/histamine complex was effective in treating CSU and concomitant panic disorder in this patient and could be effective in treating some types of panic disorder. Considering the mechanisms of action of histamine and the immunoglobulin/histamine complex together with the patient’s clinical progress, histamine seemed to be related to panic disorder in this case. The concept of histamine-mediated syndromes, including allergies and psychiatric disorders, shows that a wider disease identity may be needed. Further studies on the immunopathogenesis of panic disorder and the mechanisms of action of the immunoglobulin/histamine complex are necessary.

## Background

Panic disorder and panic attacks are two of the most common problems in psychiatry [1]. Panic disorder is characterized by recurrent, unexpected panic attacks. Panic attacks are defined by the Diagnostic and Statistical Manual of Mental Health Disorders (DSM) as “an abrupt surge of intense fear or discomfort” reaching a peak within minutes. Panic disorder is not a benign disease; it can significantly affect quality of life and lead to depression and disability.

A specific relationship between allergic diseases and panic disorder has been strongly suggested [2]. Asthma is a risk factor for the development of panic disorder [3]. The cooccurrence of panic disorder and asthma is greater than would be expected based on their individual prevalence rates. This may be due in part to the important role of respiratory factors in panic disorder. The similarities between hyperventilation syndrome and panic disorder have also been considered recently [4]. Panic disorder and asthma and their causes and effects were the focus of multiple studies [5, 6].

In 1992, Schmidt-Traub S suggested a psychoimmunological correlation between allergies and panic disorder [7]. The relationship between panic disorder and allergies/anaphylaxis (immunoglobulin E [IgE]-mediated immediate reaction) was demonstrated, and panic disorder was diagnosed as a psychoimmunological disturbance and treated with immunotherapy or hyposensitization to the allergy. Subsequently, in 1997, Schmidt-Traub S and Bamler KJ reported that 70% of patients suffering from panic disorder displayed type I immediate reactions (controls = 29%), and the association between panic disorder and allergic (vasomotor) reactions was found to be highly significant [8]. Anxiety disorder has also been reported to be associated with allergies [9].

In 2002, an association between allergic rhinitis and psychological disturbances was reported [10]. In this report, allergic conditions seemed to be related to panic disorder regardless of the presence of hyperventilation in asthma. Concomitant allergic rhinitis and panic disorder were reported in one patient [11]. More recently, allergic rhinitis was reported to increase the risk of incident panic disorder in a nationwide population-based cohort study in Taiwan [12].

The effects of allergy treatment on the improvement of panic disorder have been indirectly suggested. Allergies were associated with an increased prevalence of panic disorder as well as any anxiety disorder, any mood disorder, depression and bipolar disorder [13]. However, after adjusting for desensitization treatment status, these relationships were no longer significant. Individuals who were treated for allergies were significantly less likely to have any mood or anxiety disorder than untreated individuals.

The recommended drugs for panic disorder include selective serotonin reuptake inhibitors, tricyclic antidepressants and serotonin-norepinephrine reuptake inhibitors; for treatment-resistant patients with no history of addiction, benzodiazepines such as alprazolam may be used if tolerable [14]. Other treatment options include irreversible and reversible monoamine oxidase inhibitors, hydroxyzine, and others.

In 1995, Gupta MA and Gupta AK reported two patients with chronic idiopathic urticaria concomitantly with panic disorder [15]. Both conditions were improved through favorable responses to the selective serotonin reuptake inhibitor antidepressants fluoxetine and sertraline. These drugs only have weak antihistaminic and anticholinergic effects. Through this case report, these effects suggested a common pathogenic factor involving serotoninergic mechanisms.

Doxepin binds to the histamine H1 receptor [16]. The histamine H1 receptor is present in the brain [17]**,** and doxepin has been used as a histamine receptor antagonist for the treatment of insomnia [18]. Hydroxyzine, a first-generation histamine H1 receptor antagonist used for the symptomatic control of chronic spontaneous urticaria (CSU), was suggested as an alternative treatment option for panic disorder [14]. Additionally, it was reported that panic disorder was treated with the antihistamine chlorpheniramine [19]. In patients with CSU, the prevalence of mental disorders is very high, with severe emotional distress [20]. This may be an indicator of the relationship between allergies, including CSU, and panic disorder that can aid in the selection of treatment.

Histobulin™ (Green Cross PD, Korea) is a histamine-fixed immunoglobulin preparation (immunoglobulin/histamine complex) [21]. The immunoglobulin/histamine complex is known to be effective in treating CSU [22]. Here, we report a patient with CSU who suffered from concomitant panic disorder, and immunoglobulin/histamine complex therapy was conducted for the treatment of CSU. In this patient, panic disorder and the clinical manifestations of CSU developed at a similar time and persisted for 23 years simultaneously. At this point, it was thought that the two diseases may be related, and panic disorder symptoms were examined closely. The successful treatment of concomitant panic disorder with CSU using immunoglobulin/histamine complex is described along with the effects of immunoglobulin/histamine complex therapy on panic disorder.

## Case presentation

A 52-year-old female Korean patient visited the Department of Allergy and Clinical Immunology, Cheju Halla General Hospital, due to symptoms of itching and urticaria. She had suffered from itching and urticaria once a month for 23 years. However, the symptoms began to develop over her whole body, and she began to take hydroxyzine every day until she caught a common cold a month before her first visit. Itching and urticaria developed before the patient started taking hydroxyzine and were incompletely controlled by it. In particular, her itching and urticaria symptoms were severe during the premenstrual period.

As noted in her medical history, the patient described that she experienced the common cold with allergic rhinitis nearly every day for 10 years before her first visit. She had suffered from the common cold for more than 20 days over the past month. However, she had no history of asthma. Concerning panic disorder, she had suffered from depression for 13 years; her panic attacks were clearly developed, and she had suffered from panic disorder for more than 10 years. The clinical manifestations of both chronic urticaria and panic disorder developed in a similar period after her divorce. At the time of the first visit, she was taking 20 mg of escitalopram, 0.75 mg of alprazolam and 25 mg of imipramine daily for the clinical manifestations of panic disorder. Additionally, to control insomnia, she took 5 mg of zolpidem daily, independent of other medications. She had no other medical history, including hyperthyroidism or cardiopulmonary disorders. She had two children, one son and one daughter, and a history of artificial abortion two times after the last delivery. She had no specific medication history before urticaria and panic disorder developed. Concerning social environment, she was a housewife who did not work, and after her divorce, she worked in the laundry. She smoked 1/10 pack per day, once or twice a week for 10 years intermittently. She drank a bottle of alcohol per day twice a week for 15 years.

In her family history, her second elder sister had angioedema on the lip, and her fifth elder sister had severe depression.

In the physical examination, her blood pressure was 138/87, her pulse rate was 79 beats per minute, and her body temperature was 36.4 °C. Her breathing sounded clear without rales or wheezes. Her heartbeat was regular without murmur. The liver, kidney, spleen or any abnormal mass was not palpable. There was no neurologic sign or sensory or motor dysfunction. Her sense of balance was normal. There was no Babinski sign. In particular, there were no dermatologic findings at the first visit other than urticaria.

The patient exhibited urticaria, skin rash and itching over a period of 6 weeks. Urticaria developed without any triggering factors, such as physical pressure, cold exposure or exercise. Dermographism was present when urticaria developed. The skin rash, itching and urticaria were properly treated but not completely controlled by antihistamines (hydroxyzine, an H1 receptor blocker). According to the criteria, the patient was diagnosed with CSU [23]. Under the presumption of CSU and panic disorder, allergy tests and a psychiatric evaluation were conducted.

Basic laboratory tests, including complete blood differential counts, routine blood chemistry for liver function and renal function and urinalysis, were conducted, and there were no abnormal findings in the liver function tests, renal function tests or urinalysis before treatment (Table 1).

**Table 1:** Basic initial laboratory results, including liver function tests and renal function tests

Basic laboratory test		Results	Normal range	Unit
CBC	WBC	12,000	3900–11,000	1000/μl
	RBC	4.52	3.82–5.40	1,000,000/μl
	Hb	14.0	12.0–17.0	g/dL
	Hct	41.3	35.0–51.0	%
	Neutorphil %	54.1	39.7–73.8	%
	Lymphocyte %	37.5	18.8–49.2	%
	Monocyte %	6.6	0.0–10.0	%
	Eosinophil %	1.2	0–5	%
	Basophil %	0.6	0–1	%
Blood Chemistry	Total protein	6.8	6.6–8.7	g/dL
	Albumin	4.5	3.5–5.2	g/dL
	Alkaline Phosphatase	72	35–130	U/L
	AST (GOT)	17	0–40	U/L
	ALT (GPT)	13	0–41	U/L
	Glucose	90	70–110	mg/dL
	Uric acid	2.7	2.4–5.7	mg/dL
	BUN	8	6–20	mg/dL
	Creatinine	0.7	0.5–0.9	mg/dL
	Calcium	9.3	8.6–10.2	mg/dL
	Phosphorus	4.0	2.5–4.5	mg/dL
	Na	140	136–145	mEq/L
	K	4.6	3.5–5.1	mEq/L
	Cl	102	98–110	mEq/L

WBC, White Blood Cell; RBC, Red Blood Cell; Hb, Hemoglobin; Hct, Hematocrit; AST, Aspartate Transaminase; GOT, Glutamic Oxaloacetic Transaminase; ALT, Alanine Transaminase; GPT, Glutamic Pyruvic Transaminase; BUN, Blood Urea Nitrogen

Basic allergy tests, including serum eosinophil cationic protein test, serum total IgE and IgE levels for specific allergens using a multiple allergosorbent test (MAST, Green Cross PD, Korea) and skin prick test, were conducted before and after treatment. In the MAST, the specific IgEs for 41 allergens were evaluated, including the following: *Dermatophagoides pteronyssinus* (Dp), *Dermatophagoides farina* (Df), cat, dog, egg white, milk, soybean, crab, shrimp, peach, mackerel, rye pollen, house dust mite, cockroach, *Clasporium herbarum, Aspergillus fumigatus, Alternaria alternata*, birch-alder mix, white oak, short ragweed, mugwort, Japanese hops, hazelnut, sweet vernal grass, Bermuda grass, orchard grass, timothy grass, reed, *Penicillium notatum*, sycamore, sallow willow, poplar mix, ash mix, pine, Japanese cedar, acacia, oxeye daisy, dandelion, Russian thistle, goldenrod and pigweed. The test results showed the level of specific IgE for each allergen, and a normal negative range was 0.000–0.349 IU/ml.

A skin prick test was also performed for 53 allergens. The allergens tested were as follows: *Alternaria alternata, Aspergillus fumigatus, Aspergillus nigre, Candida albicans, Cladosporium, Penicillium chrysogenum*, German cockroach, Dp, Df, dog, cat, gray elder/silver birch, grass mix, mugwort, short ragweed, black willow pollen, orchard grass, Bermuda grass, timothy grass, English plantain, English rye grass, Holm oak, Japanese cedar, cotton flock, milk mix, egg mix, chicken, beef, pork, cod, oyster, salmon, prawn, mackerel, tuna, almond, peanut, bean, carrot, cabbage, walnut, maize, peach, tomato, black pepper, spinach, wheat flour, rabbit, kapok, hops, acacia, pine and poplar. Skin prick tests were performed on the patient’s back. The area to be tested was cleaned with alcohol and coded with a skin marker corresponding to the number of allergens being tested. The marks were 2 cm apart. A drop of allergen solution was placed beside each mark. A small prick was made through the allergen solution drop into the skin using a Morrow Brown needle (Morrow Brown Allergy Diagnostics, USA) by holding the needle perpendicular to the test site and punching firmly through the test extract and into the epidermis. The drop was removed immediately after the skin was pricked, and the used needle was discarded immediately. Histamine hydrochloride (10 mg/ml) was used as a positive control, and physiological saline was used as a negative control. The results were determined according to wheal size. Reactions were read after 15 min and described as negative (0, no reaction), 1+ (reaction greater than the control reaction but smaller than half the size of the reaction to histamine), 2+ (equal to or more than half the size of the histamine reaction), 3+ (equal to or more than the size of the histamine reaction) and 4+ (equal to or more than twice the size of the histamine reaction). The minimum size of a positive reaction was 3 mm.

The clinical severity of CSU was evaluated using the urticaria severity score by Jariwala *et al.* [24]. Of the 12 questions, 10 were scored on a scale of 0 to 7, with higher scores reflecting greater severity of symptoms; increased disruption of quality of life components, including sleeping, work/school attendance, and social life; and increased amounts and use of antihistamine or oral corticosteroid medications. Two questions involved the body areas showing symptoms and had 8 answer choices per question; increased scores suggested more extensive urticaria symptoms. One of the questions was about the amount of oral corticosteroids used; this question was double-weighted, and the item score was multiplied by 2, considering that the need for oral corticosteroids reflected an increased severity of disease. However, steroids were not used in this report. The urticaria severity score was calculated by adding the score for each question, resulting in a maximum score of 93 and a minimum score of 0.

For panic disorder, the patient was evaluated by extensive diagnostic interviews at baseline by a psychiatrist. The diagnostic interviews systematically assessed the diagnostic criteria outlined by the DSM-V [25]. The patient was evaluated by a clinical psychologist at the Institute of Clinical Psychology before, during and after treatment to determine her mood using the Beck Depression Inventory (BDI-2) for depression (normal ≤ 13, mild 14–19, moderate 20–28, and severe 29–63), the State-Trait Anxiety Inventory (STAI) for anxiety (state: normal ≤ 51, mild 52–56, moderate 57–61, and severe 62–80; trait: normal ≤ 53, mild 54–58, moderate 59–63, and severe 64–80) and the Beck Hopelessness Score for hopelessness (normal < 4, mild 4–8, moderate 9–14, and severe 15–20) [26, 27].

The initial clinical severity of the patient’s CSU was 41 points and was evaluated at every visit. In particular, in the initial laboratory tests for allergic evaluation before treatment, the white blood cell (WBC) count was 12,000 (3900–11,000 count/mm^3^), the eosinophil fraction was 1.2 (0–5%), and the basophil fraction was 0.6 (0–1%). The blood eosinophil cationic protein level was 9.66 (0–24 ng/ml), and the serum total IgE level was 30.8 (0–350 IU/ml). To rule out selective IgA deficiency for immunoglobulin/histamine complex therapy, IgA levels were measured; the result was 64.2 (70–400 mg/dL), which was slightly low. In the follow-up laboratory test after treatment, the WBC count was 6180 (3900–11,000 count/mm^3^), eosinophil fraction was 3.2 (0–5%) and basophil fraction was 0.6 (0–1%). The blood eosinophil cationic protein level was 4.96 (0–24 ng/ml), and the serum total IgE level was 28.3 (0–350 IU/ml). In the MAST, a specific IgE was detected only for Df, with a measurement of 0.6 (0–0.49 IU/ml) before treatment and 0.47 after treatment. In the skin prick test, the patient did not respond to any tested allergens before or after treatment.

At the time of the first diagnosis of panic disorder, the patient exhibited recurrent unexpected panic attacks (Table 2). A panic attack is an abrupt surge of intense fear or intense discomfort that reaches a peak within minutes and persists, possibly for an entire day, and includes the following symptoms: palpitations, sweating, trembling of the body, sensations of shortness of breath or being smothered, chest pain or discomfort, nausea, paresthesias (tingling sensations of both fingers and toes), fear of losing control, fear of dying and severe headache. Additionally, she had persistent concern or worry about additional panic attacks or their consequences nearly every time after a panic attack. She took no medications and had no systemic diseases. Therefore, these disturbances were not attributable to the physiological effects of a substance or another medical condition. After the first panic attack, she was diagnosed with depression because the disturbance was not better explained by another mental disorder, such as social anxiety disorder, specific phobia, obsessive‐compulsive disorder, posttraumatic stress disorder or separation anxiety disorder. Although the symptoms and signs developed after her divorce, it was not determined that the clinical manifestations were attributed to the divorce, and posttraumatic stress disorder was ruled out. At the time of her first visit just before treatment, only the symptom of sweating disappeared after panic attacks. She still felt the sensations of shortness of breath or being smothered. In particular, she experienced respiratory difficulty during inhalation. With hyperventilation due to respiratory difficulty, she experienced fainting once at the time of her first diagnosis. She also suffered from severe insomnia, and she described that the severity and frequency of the panic attacks seemed to be related to the degree of insomnia.

**Table 2:** Psychiatric symptoms and signs of the patient aligned with the DSM-V criteria for panic disorder

Dx categories	Symptoms during the 1 month	At the time of Dx	Before Tx	After Tx
(A) Recurrent unexpected panic attacks. A panic attack is an abrupt surge of intense fear or intense discomfort that reaches a peak within minutes, and during which time four (or more) of the following symptoms in right column occur	Palpitation	O	O	X
	Pounding heart	X	X	X
	Sweating	O	X	X
	1) Trembling or 2) shaking	O (1)	O (1)	X
	1) Sensations of shortness of breath or 2) smothering	O (both)	O (both)	X
	Feeling of choking	X	X	X
	1) Chest pain or 2) discomfort	O (both)	O (both)	
	1) Nausea or 2) abdominal distress	O (1)	O (1)	X
	1) Feeling dizzy, 2) unsteady, 3) light-headed, or 4) faint	X	X	X
	1) Chills or 2) heat sensations	X	X	X
	Paresthesias (1) numbness or 2) tingling sensations)	O (2)	O (2)	X
	1) Derealization (feelings of unreality) or 2) depersonalization (being detached from oneset, 3) fear of losing control or 4) “going crazy”	O (3)	O (3)	X
	Fear of dying	O	O	X
(B) At least one of the attacks has been followed by 1 month (or more) of one or both of the following in the right column	Persistent concern or worry about additional panic attacks or their consequences (for example, losing control, having a heart attack, “going crazy”)	O	O	O
	A significant maladaptive change in behavior related to the attacks (for example, behaviors designed to avoid having panic attacks, such as avoidance of exercise or unfamiliar situations)	X	X	X

According to the diagnostic criteria of the DSM-V, she met the criteria for panic disorder at the time of the first diagnosis ten years prior and before immunoglobulin/histamine complex therapy. In addition to the medication for panic disorder, she took sleeping pills for severe insomnia every day before treatment.

Immunoglobulin/histamine complex therapy was started for the treatment of CSU. The patient received 2 ml of Histobulin™ (12 mg human immunoglobulin/0.15 µg histamine complex) once a week by subcutaneous injection in the deltoid area of the upper arm as described in a previous report [28]. She was instructed to take a tablet of 5 mg levocetirizine if necessary when she felt uncomfortable due to urticaria, skin rash or itching that prevented her from engaging in activities of normal daily living. For the panic disorder, she was instructed to take 20 mg of escitalopram, 0.75 mg of alprazolam and 25 mg of imipramine when she felt the clinical manifestations of panic disorder. She was instructed to take 5 mg of zolpidem to control insomnia. The severity of panic disorder and insomnia was represented and monitored by the frequency of medication and the appearance of clinical manifestations of panic attacks and insomnia. Immunoglobulin/histamine complex therapy was well tolerated, and there were no side effects.

**Fig. 1:**
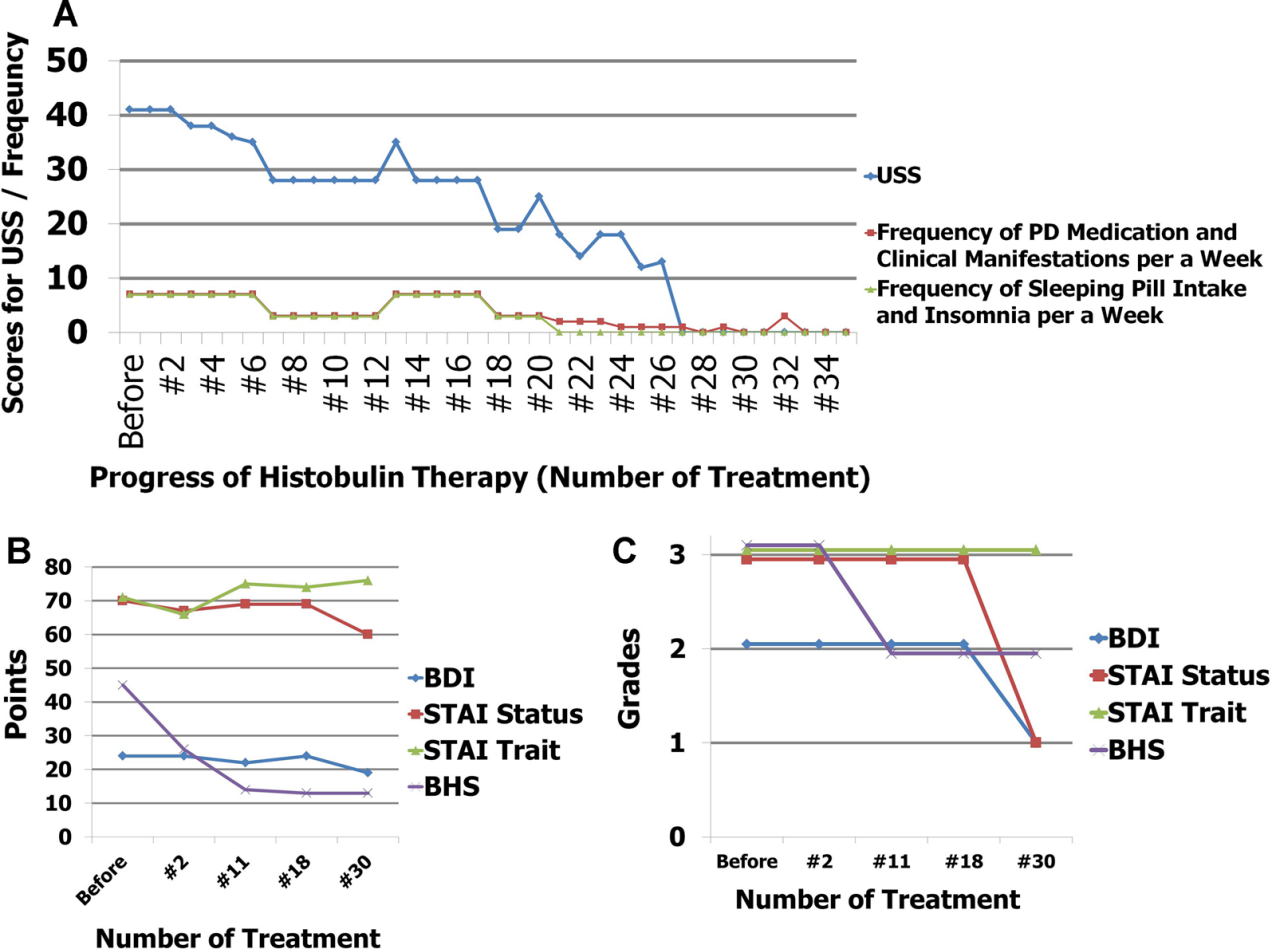
**A** Clinical progress of Histobulin™ therapy. The urticaria severity score was evaluated every week. Medication frequency was used to indicate the progression of panic disorder and insomnia. The medication frequency for panic disorder and insomnia was parallel to the severity of CSU. Additionally, the clinical manifestations and insomnia were absent despite the cessation of relevant medication. The remission of panic disorder and insomnia was achieved nearly simultaneously with the remission of CSU. Blue line: urticaria severity score during a week; red line: medication frequency for panic disorder during a week; green line: medication frequency of sleeping pills during a week. **B** Total score of psychometric measures. Psychometric indicators showed significant reductions. Blue line: urticaria severity score during a week; red line: medication frequency for panic disorder during a week; green line: medication frequency of sleeping pills during a week. **C** Grades of psychometric measures. Grades of psychometric indicators were also significantly reduced: 0, normal range; 1, mild; 2, moderate; 3, severe; blue line, BDI; red line, STAI state; green line, STAI trait; violet line, BHS

For chronic urticaria, initial improvement was achieved after the third injection (Fig. 1). The clinical severity of itching and urticaria began to decrease subjectively and objectively. The medication frequency began to decrease after the tenth injection. After the twenty-seventh injection of immunoglobulin/histamine complex, she showed no more symptoms of itching and urticaria and ceased medication. Symptom-free therapy was continued without medication for more than 4 weeks, and remission of chronic urticaria was determined after thirty-one injections. After achieving remission of CSU, her allergic rhinitis was also completely resolved. The frequency of the common cold significantly decreased to once a year during and after treatment.

The clinical course of panic disorder was similar to that of CSU during immunoglobulin/histamine complex therapy. The frequency of medication and the development of the clinical manifestations of panic disorder changed in parallel with the clinical severity of CSU. Moreover, the patient did not experience clinical manifestations of panic disorder and ceased both the medication for panic disorder and sleeping pills for insomnia simultaneously with the remission of CSU (Fig. 1A). The reduced frequency and duration alleviated her persistent concern or worry about additional panic attacks and their consequences, but a small amount of concern and worry remained due to her past experiences. In the psychological evaluation, the BDI, STAI and BHS scores were also improved with improvement of panic disorder as well as CSU (Fig. 1B and C).

## Discussion and conclusions

In this patient, panic disorder was treated successfully using the immunoglobulin/histamine complex with the simultaneous remission of CSU (Fig. 1). The occurrence and clinical progress of panic disorder were parallel to those of CSU. At the time of this report, there have been no further clinical manifestations of panic disorder without medication for more than 2 years. This is the first report to describe the treatment of panic disorder with immunoglobulin/histamine complex, an agent typically used for allergy treatment.

The patient suffered from panic disorder and allergic disease for more than 23 years, and the results of this successful treatment for panic disorder are not considered a natural cure. With the remission of CSU, allergic rhinitis was completely improved, and the frequency of upper respiratory infections was significantly reduced.

The clinical manifestations of chronic urticaria developed in a similar period as the panic disorder. During the treatment, the CSU severity score was parallel to the frequency of medication and clinical manifestations of panic disorder (Fig. 1A). Psychometric measures, including those for depression and anxiety, showed significant reductions in total scores with a pronounced decline in the number and intensity of panic attacks. These results suggested that the panic disorder was related to the progression of CSU.

Histamine plays a role in the balance of neuroimmunomodulation [29]. Stress induces an imbalance of neuroimmunomodulation that involves the immune, central nervous and endocrine systems. Receptors for substances involved in stress reactions, including histamine, are present in the immune system, and the peripheral, central, cortical and subcortical nervous structures influence the immune response. Histamine H1 receptor antagonists are considered for the treatment of psychiatric disorders, including panic disorder [14]. Mediated by histamine, the central nervous system and immune system can possibly affect each other, which presents as panic disorder and CSU through neuroimmunomodulation.

Hydroxyzine, a first-generation histamine H1 receptor antagonist, was used for symptomatic control for this patient before immunoglobulin/histamine complex therapy. Hydroxyzine has been suggested as an alternative therapy for panic disorder [14]. There have been several reports of panic disorder treated with the histamine H1 receptor antagonist chlorpheniramine [19, 30, 31]. These reports suggested that the effect of chlorpheniramine on panic disorder was due to its serotonin reuptake-inhibiting property [32]. In this patient, not only was the symptomatic control of CSU incomplete with the histamine H1 antagonist hydroxyzine, but there was also no improvement in the clinical progress of her panic disorder for 23 years. However, with immunoglobulin/histamine complex therapy, panic disorder and CSU were remitted, and the patient showed no further clinical manifestations of panic disorder and CSU without medication. The mechanisms of action of the immunoglobulin/histamine complex have not been proven until now, but it does not appear to have serotonin reuptake-inhibitory properties.

The clinical manifestations of chronic urticaria, allergic rhinitis and panic disorder in this patient developed within a similar period. During the treatment, the clinical courses for CSU and panic disorder were nearly parallel to each other. Moreover, remission of both CSU and panic disorder was achieved in a similar period. Considering these clinical results, the immunopathogenesis of panic disorder and CSU, as well as allergic rhinitis, seemed to be similar, and histamine appeared to be the key for the immunopathogenesis of both diseases, considering the histaminopexy effects of the immunoglobulin/histamine complex.

Antihistamine H1 therapy blocks only the H1 receptor and interferes with the action of histamine through this receptor [33]. Four types of histamine receptors (H1, H2, H3 and H4) are present in the brain [34]. The representative mechanism of action of the immunoglobulin/histamine complex is histaminopexy [35]. This effect is similar to the effect of a histamine H1 receptor antagonist, which interferes with the action of histamine. Moreover, this mechanism of action may affect all four types of histamine receptors by reducing histamine levels. Panic disorder in this case did not seem to be improved by blocking only the H1 receptor and instead could possibly be resolved by the immunoglobulin/histamine complex, which might reduce the levels of histamines that affect all 4 histamine receptors. This result suggests that histamine is involved in nervous system disorders, such as panic disorder, through H2, H3, and/or H4 receptors, as expected. Considering the histaminopexy effects, in this case, histamine seemed to participate in the immunopathogenesis of panic disorder or a subcategory of panic disorder.

Most importantly, remission of panic disorder and CSU was induced. With the simple histaminopexy effect, the clinical improvement is transient, and remission cannot be induced. It is also very important to understand the immunopathogenesis of panic disorder in terms of the role of histamine. The immunoglobulin/histamine complex was reported to induce antihistamine antibodies, which might have persistent histaminopexy effects that may reduce or normalize blood histamine levels [36, 37].

Histaminopexy is the mechanism of action of the immunoglobulin/histamine complex, and from the results in this case, several possibilities could be deduced. 1) CSU may be the key triggering factor for panic disorder, or 2) the clinical manifestations of panic disorder, including panic attacks, may be provoked by histamine through neuroimmunomodulation, making histamine the main pathogenetic mediator of panic disorder, and CSU is accompanied by panic disorder. The two diseases together seem to constitute histamine-mediated syndrome (HMS), a syndrome caused by the actions of histamine through neuroimmunomodulation, in which the effects of histamine are imbalanced according to the sensitivity of histamine receptors (histamine receptor sensitivity imbalance).

**Fig. 2:**
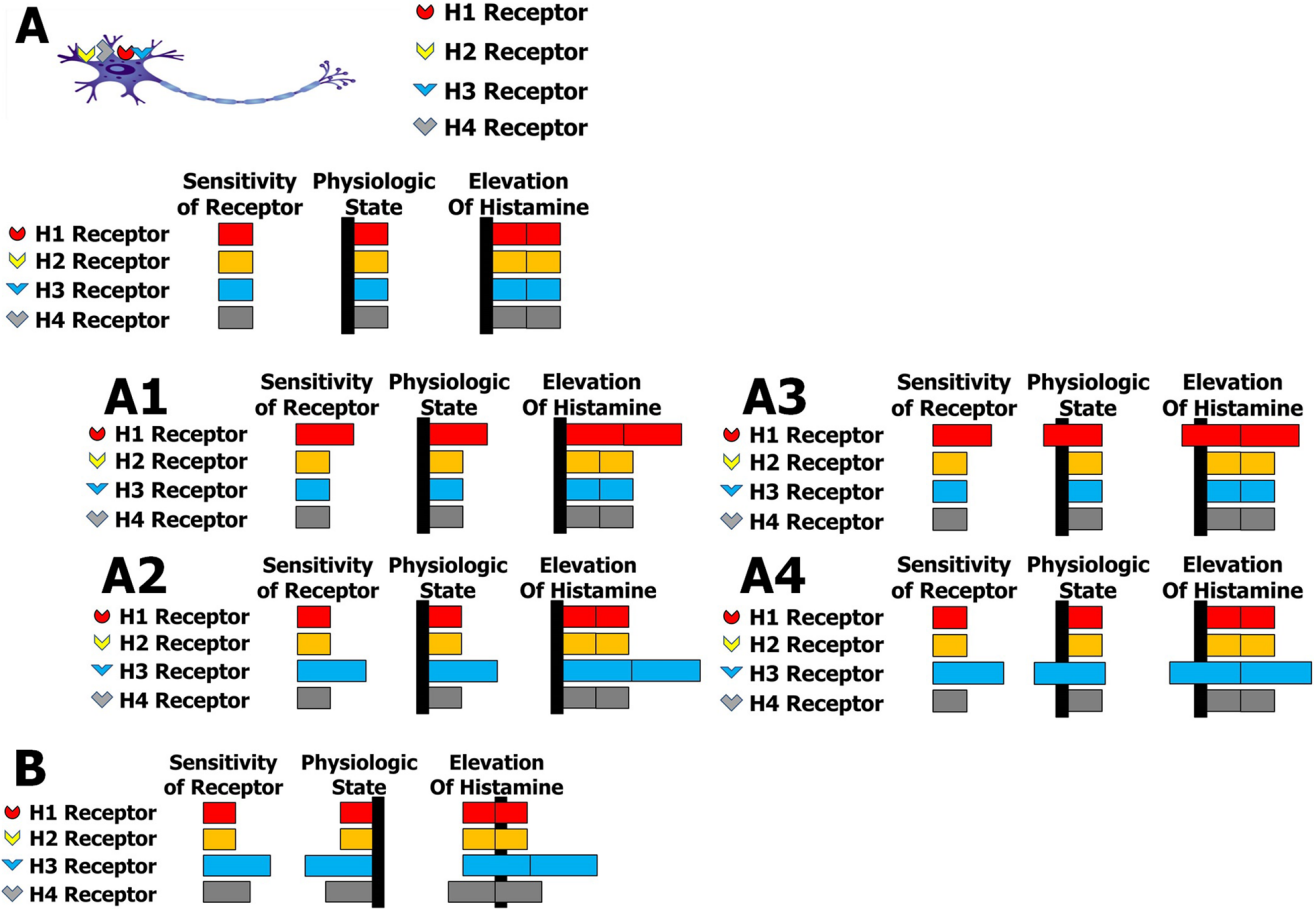
Histamine receptor sensitivity balance theory. The hypothesis is that histamine is a common mediator of CSU and panic disorder. Four types of histamine receptors are present in neurons: H1, H2, H3 and H4. This theory involves the hypothesis that the sensitivity of the four neuron receptors to histamine can be the same or different for many reasons, and two possibilities may be present. **A** The first possibility presupposes that the normal status is when the sensitivities of the four receptors are equal. Therefore, the responses of receptors to histamine are equivalent, regardless of the level of histamine (2A). Under normal conditions, patients may exhibit urticaria without other neurological symptoms and signs despite elevated histamine levels while maintaining the balance in their neuron responses. If a constitutional imbalance in the sensitivity of histamine receptors is present, the clinical manifestations of neurological, psychological and/or psychiatric disorders (constitutional histamine-mediated syndrome) may develop (**2A1** and **2A2**). The possible kinds of sensitivity imbalance may occur in many cases due to the combination of receptor types. The clinical manifestations may differ according to the sensitivity among the receptors. Additionally, subjects can possibly adapt to a balanced state among the responses of the receptors to histamine in the normal state (**2A3** and **2A4**). However, if histamine levels are increased due to pathological conditions, such as allergic responses, anaphylaxis, allergic rhinitis, asthmatic attacks and/or chronic urticaria, the clinical manifestations of neurological, psychological and/or psychiatric disorders may develop due to the unbalanced responses of the four types of neuron histamine receptors (induced histamine-mediated syndrome). Histamine receptor sensitivity may be related to many conditions, including the number of receptors on target cells and gene mutation of the receptors. **B** The other hypothesis is that the normal status is unequal sensitivities of the four receptors (**2B**). At the physiological level, the responses of receptors to histamine maintain normal physiological conditions. However, when the histamine level is increased, neurological and psychological symptoms and signs appear. However, these neurological and psychological changes may be normal defense mechanisms. Namely, if a situation such as anaphylaxis occurs and urticaria develops, the subject might focus on their defense response. Therefore, the subject becomes nervous in situations in which anxiety and panic feel like defense mechanisms. Therefore, it may be natural that subjects experience anxiety and have feelings of panic in situations such as anaphylaxis where histamine levels are raised

The histamine receptor sensitivity balance theory was suggested, and two hypotheses are possible. (1) Histamine is a common mediator of CSU and panic disorder, and (2) the sensitivity of the four neuron receptors to histamine can be the same or different for many reasons. Two possibilities may be present. The first presupposes that the normal status is that the sensitivities of the four receptors are equal. Therefore, the responses of receptors to histamine are equivalent regardless of the level of histamine (Fig. 2A). Under normal conditions, patients may show urticaria without other neurological symptoms and signs despite elevated histamine levels while maintaining the balance in their neuron responses (Fig. 2A). If a constitutional imbalance in the sensitivity of histamine receptors is present, the clinical manifestations of neurological, psychological and/or psychiatric disorders (constitutional histamine-mediated syndrome) may develop (Fig. 2A1 and A2). There are many possible variations in sensitivity imbalance due to the combination of receptor types. According to the differences in sensitivity among the receptors, the clinical manifestations may differ. Additionally, subjects may have adapted a balanced state among the responses of the receptors to histamine in the normal state (Fig. 2A3 and A4). However, if histamine levels are increased due to pathological conditions, such as allergy provocation, anaphylaxis, allergic rhinitis, asthmatic attacks and/or chronic urticaria, the clinical manifestations of neurological, psychological and/or psychiatric disorders may develop due to the unbalanced responses of the four types of histamine receptors (induced histamine-mediated syndrome). Histamine receptor sensitivity may be related to many conditions, including the number of receptors on target cells and gene mutations of the receptors.

In acute conditions such as anaphylaxis and asthmatic attacks, the raised histamine level is transient; the clinical manifestations of neuropsychological and psychiatric disorders may also be transient and may possibly improve with the normalization of the histamine concentration to the physiological level with the resolution of allergy responses. However, neuropsychological and psychiatric disorders persist in chronic allergic conditions, and histamine levels are maintained or increased persistently in conditions such as chronic urticaria, perennial allergic rhinitis or persisting asthmatic conditions, as in this case.

According to this hypothesis, a histamine receptor 1 antagonist may be effective when histamine receptor 1 (H1) is more sensitive to histamine than the H2, H3, and H4 receptors. Not only was hydroxyzine suggested as an alternative therapeutic option for panic disorder [14], but there have been several reports where panic disorder was treated with the histamine H1 receptor antagonist chlorpheniramine [19, 30–32]**,** as described above (Fig. 2A1 and A3). According to the hypothesis, the case in this report may include the conditions that are shown in Fig. 2A4 and B.

The second possibility presupposes unequal sensitivities of the four receptors (Fig. 2B). At the physiological level, the responses of receptors to histamine maintain normal physiological conditions. However, when the histamine level is increased, neurological and psychological symptoms and signs appear. However, these changes may be normal defense mechanisms. Namely, if a situation such as anaphylaxis occurs and urticaria develops, the subject might concentrate on their defense response. Therefore, the subject becomes nervous in situations in which anxiety and panic feel like defense mechanisms. Therefore, it may be natural that subjects experience anxiety and have feelings of panic in situations such as anaphylaxis where histamine levels are raised.

To date, there have been no approved therapeutics for histamine receptor 3 and 4 antagonists. The immunoglobulin/histamine complex has histaminopexy effects that can reduce and normalize histamine levels. Therefore, the immunoglobulin/histamine complex currently seems to be the only simple and effective therapeutic way to control blood histamine levels. Otherwise, it is necessary to calibrate the response of all histamine receptors using H1, H2, H3 and/or H4 blockers in detail and complexly to clinically control histamine responses. However, any specific symptoms and signs may be more easily controllable using one or two antagonists for one or two histamine receptors. To control allergic symptoms and signs, only histamine H1 receptor blockers may be needed. However, for the control of HMS, which is mediated through the histamine receptors H1, H2, H3 and/or H4, specific blockers may be needed. Therefore, the immunoglobulin/histamine complex can treat all the symptoms and signs mediated by raised histamine levels, as in this case. According to the hypothesis, the case in this report may be a chronic condition, as shown in Fig. 2A4 and B. According to the histamine receptor sensitivity balance theory, many kinds of pathological conditions can occur due to the many combinations of unbalanced receptor sensitivity among the four receptors.

Another possibility is that panic disorder and chronic urticaria are independent diseases and do not share an immunopathogenesis. However, they were improved by the same mechanisms of action of the immunoglobulin/histamine complex, which has several immunomodulation mechanisms other than histaminopexy. The main constituent of the immunoglobulin/histamine complex is immunoglobulin, and the immunoglobulin/histamine complex may be used as a small dose of intravenous immunoglobulin (IVIG). IVIG has immunomodulatory effects, especially anti-autoimmune effects [38, 39]. Considering the autoimmune characteristics of chronic urticaria and the effects of IVIG on autoimmune chronic urticaria, the immunoglobulin/histamine complex may be effective in chronic urticaria and the immunopathogenesis of autoimmune diseases [40]. The relationship between autoimmune diseases and panic disorder has been reported previously [41–43]**,** and the possibility was raised that autoimmune mechanisms could interrupt neurotransmission, which would be of significance in certain patients with anxiety and panic disorders [44]. Considering these aspects, panic disorder and CSU can be treated with an immunoglobulin/histamine complex with the anti-autoimmune effects of a small quantity of IVIG. By this explanation, CSU and panic disorder may be different disease entities merely with similar autoimmune mechanisms.

The immunoglobulin/histamine complex was reported to be effective in allergic rhinitis and chronic urticaria. In this case, allergic rhinitis was completely improved with the remission of CSU. In a previous report, multiple allergic diseases were improved simultaneously using the immunoglobulin/histamine complex [45]. The immunoglobulin/histamine complex seems to solve allergic conditions at the basic level of allergy immunopathogenesis. Additionally, in this report, the frequency of upper respiratory infection was significantly decreased during and after treatment with the immunoglobulin/histamine complex, similar to previous reports [28, 45]. Considering the current severe acute respiratory syndrome coronavirus-2 (SARS CoV-2) pandemic, as well as the SARS CoV 1 virus and the Middle East respiratory syndrome coronavirus (MERS-CoV), the preventive and/or therapeutic effects of the immunoglobulin/histamine complex in upper respiratory infections may be notable for the current pandemic, and other new viral infections may appear in the future.

From the results, the concept of the disease entity known as HMS, which includes other clinical manifestations that can be provoked by histamine, such as psychiatric disorders and allergic diseases, through neuroimmunomodulation seems to be necessary. From the viewpoint of allergies, clinical manifestations of psychiatric disorders may be para-allergic diseases considering mediation by histamine. Additionally, further efforts to investigate the disease spectrum that can be provoked by histamine, other than allergies, are absolutely necessary.

Conclusively, the immunoglobulin/histamine complex seems to be effective in treating panic disorder, as well as CSU, at least in this case. There is a possibility that histamine participates in the immunopathogenesis of panic disorder considering the action mechanisms of the immunoglobulin/histamine complex. HMS and the subsequent histamine receptor balance theory were suggested for the development of psychiatric and neurological diseases. The concept of HMS, including the clinical manifestations of allergic diseases, psychiatric disorders and other organs that can be affected by histamine, may be needed as a wider disease identity. Additionally, further studies on the immunopathogenesis of panic disorder and the mechanisms of action of the immunoglobulin/histamine complex may be necessary. As reported previously, the immunoglobulin/histamine complex seems to be effective for the simultaneous treatment of multiple allergic diseases and for the prevention of upper respiratory infections and should be studied for its preventive effects in the pandemic period on serious viral infections, including SARS-CoV-2.

## Data Availability

Not applicable.

## Abbreviations

CU: Chronic urticaria
CSU: Chronic spontaneous urticaria
Dp: Dermatophagoides pteronyssinus
Df: Dermatophagoides farina
BDI: Beck Depression Inventory
STAI: State-Trait Anxiety Inventory
BHS: Beck Hopelessness Score
HMS: Histamine-mediated syndrome
IVIG: Intravenous immunoglobulin
SARS CoV-2: Severe acute respiratory syndrome coronavirus-2
MERS-CoV: Middle East respiratory syndrome coronavirus
